# Synergistic effect of pyrvinium pamoate and posaconazole against *Cryptococcus neoformans in vitro* and *in vivo*

**DOI:** 10.3389/fcimb.2022.1074903

**Published:** 2022-12-09

**Authors:** Yali Li, Sheng Li, Min Chen, Jialing Xiao, Hong Fang

**Affiliations:** ^1^ Department of Dermatology, The First Affiliated Hospital, School of Medicine, Zhejiang University, Hangzhou, China; ^2^ Department of Dermatology, Shanghai Changzheng Hospital, Shanghai, China; ^3^ Department of Stomatology, Zhejiang Hospital, Hangzhou, China

**Keywords:** *cryptococcus neoformans*, pyrvinium pamoate, azoles, antifungal, synergistic effect, posaconazole

## Abstract

**Background:**

Cryptococcosis is a global invasive mycosis with high rates of morbidity and mortality, especially in AIDS patients. Its treatment remains challenging because of the limited antifungals and their unavoidable toxicity, and as such more efforts need to focus on the development of novel effective drugs. Previous studies have indicated that pyrvinium pamoate (PP) has individual and synergistic fungistatic effect. In this study, the effects of PP alone and in combination with azoles [fluconazole (FLU), itraconazole (ITR), voriconazole (VOR), posaconazole (POS)] or amphotericin B (AmB) were evaluated against *Cryptococcus neoformans* both *in vitro* and *in vivo*.

**Methods:**

A total of 20 *C. neoformans* strains collected from cryptococcal pneumonia and cryptococcal meningitis were studied. The effects of PP alone, PP-azoles and PP-AmB interactions against *C. neoformans* were evaluated *via* the microdilution chequerboard technique, adapted from broth microdilution method according to the CLSI M27-A4. The *in vivo* antifungal activity of PP alone and in combination with azoles and AmB against *C. neoformans* infections was evaluated by *Galleria mellonella* survival assay.

**Results:**

The *in vitro* results revealed that PP individually was ineffective against *C. neoformans* (MIC>16 μg/ml). Nevertheless, the synergistic effects of PP with ITR, VOR, POS, FLU or AmB was observed in 13 (65.0%, FICI 0.188–0.365), 3 (15.0%, FICI 0.245-0.301), 19 (95.0%, FICI 0.188-0.375), 7 (35.0%, FICI 0.188-0.375), and 12(60.0%, FICI 0.281-0.375) strains of *C. neoformans*, respectively. There was no antagonism. The survival rates of larvae treated with PP (3.33%) showed almost no antifungal effective, but the larvae survival rates improved when PP combined with AmB (35% vs. 23.33%), FLU (40% vs. 25%), ITR (48.33% vs. 33.33%), VOR (48.33% vs. 53.33%) and POS (56.67% vs. 36.67%) comparison with AmB or azoles alone, and statistical significance was observed when PP combined with POS versus POS alone (*P* = 0.04).

**Conclusions:**

In summary, the preliminary results indicated the potential of PP in reduction the MICs of azoles and AmB, also itself against *C. neoformans*; the combination of PP with AMB, FLU, ITR, VOR and POS improve the survival rates of *C. neoformans* infection larvae, compared with they are alone. The *in vitro* and *in vivo* data show that PP could enhance the activity of POS against *C. neoformans.* This study contributes with data of PP in combination with classical drugs of choice for cryptococcosis treatment.

## Introduction

*Cryptococcus neoformans* is an important pathogenic fungal species that cause one of the mainly opportunistic mycoses cryptococcosis, which involved multiple invasive fungal diseases of the human body including the cryptococcal meningitis, cryptococcemia and cutaneous disseminated cryptococcosis (Rajasingham et al., 2017; Centers for Disease Control and Prevention, 2022). However, in recent years, the incidence of cryptococcosis elevated more than 223,000 cases and 181,100 deaths worldwide per year, and mainly because of the immunodeficiency populations increasing (Mirza et al., 2003; Rajasingham et al., 2017). This has been accompanied by a high mortality rate, especially among with HIV -AIDS patients, where data show that 1-year mortality rates for HIV-infected cryptococcal meningitis patients ranges from 50% to 100% in low-resource settings compared to 10% to 30% in resource-rich countries (Loyse et al., 2013; Williamson et al., 2016; Rajasingham et al., 2017). The reasons for the different mortality involved in the severity of the infection, the status of host immunity and the availability of antifungal (Bermas and Geddes-McAlister, 2020).

Antifungal drugs for treatment of cryptococcosis currently is largely limited to fluconazole (FLU), amphotericin B (AmB) in combination with FLU or 5-fluorocytosine (5FC) (Perfect et al., 2010; Grossman and Casadevall, 2017). However, the adverse effects of antifungal drugs and the resistance of fungi have become two problems that need attentions in the treatment of cryptococcosis (Bermas and Geddes-McAlister, 2020). The adverse events, the cost and unlicensed limit the widespread use of AmB (Longley et al., 2013). Although the combination of AmB and 5FC has becoming a first- line induction treatment for cryptococcal meningitis with effective and synergistic interactions, however, the 5FC is unavailable in most of Asia and Africa because of lacking of manufacturers, and the cost and bone marrow toxicity, as well as four times daily dosing resulting in more noncompliance (Loyse et al., 2013; Bermas and Geddes-McAlister, 2020). Study indicated that *Cryptococcus* is resistant to FLU up to 10.6%, and the relapse isolates have higher rates up to 24% (Bongomin et al., 2018). *Cryptococcus* isolates exhibits resistance to azole antifungals, especially, an increase of FLC resistance is prevalent across the globe (Pfaller et al., 2009; Bermas and Geddes-McAlister, 2020), and the intrinsic resistance to echinocandins which may be related to the calcineurin pathway and cytoplasmic calcium homeostasis regulated by CRM1 and CDC50, and the protection of melanins (Arastehfar et al., 2020; van Duin et al., 2002; Cao et al., 2019). Thus, the search for new antifungal targets or combinations to improve the efficacy and reduce the toxicity might be a promising option, scientists have attempted to use established, FDA-approved drugs, to help devise appropriate treatment options.

Pyrvinium pamoate (PP) as a classical anthelmintic drug, was approved by FDA used for the treatment of pinworms in humans dating back to the first FDA approval for treatment of enterobiasis in 1959 (Beck et al., 1959; Fozard, 1978). Previous studies have shown PP has fungistatic, and the underlying mechanisms were that PP may toward the aneuploidy-related azole resistance in the *Candida albicans* and *Aspergillus fumigatus*; and interfere the fungal biological processes in *Exophiala dermatitidis* (Chen et al., 2015; Gao et al., 2018; Sun et al., 2020a; Sun et al., 2020b). In consideration of the importance of aneuploidy among the *C. neoformans* azole sensitivity, we speculate that PP might also exert some antifungal effect and positive interactions with conventional antifungals against *C. neoformans* (Kwon-Chung and Chang, 2012; Yang et al., 2021). In the present study, the antifungal efficacy of PP alone and in combination with triazoles and AmB against *C. neoformans* were investigated both *in vitro* and *in vivo*.

## Materials and methods

### Fungal strains, antifungals, and chemical agents

All 20 C*. neoformans* isolates were obtained from patients with clinically confirmed cryptococcosis disease. Z1-Z3, 08061, 05338 were obtained from patients with cryptococcal pneumonia. G5-G10, 05781, 07406, 07109, 07394 were obtained from patients with cryptococcal meningitis. 07764, 07789, 08026 were isolated from the blood of patients with cryptococcal pneumonia, and all isolates were characterized microscopically and molecularly according to URA5 RFLP analysis (Meyer et al., 2003). *Candida parapsilosis* ATCC 22019 and *Candida krusei* ATCC 6258 was used as a control strain for susceptibility testing in this study. Fungal isolates were required to be cultured on potato dextrose agar medium (Haibo Biotechnology Co., Ltd.) at 35°C for 3 days before susceptibility testing.

All drugs, including itraconazole (ITR) (Lot No. s2476), voriconazole (VOR) (Lot No. s1442), posaconazole (POS) (Lot No., s1257), FLU (Lot No. s1331), PP (lot No.5816), and AmB (lot No.1636) were both purchased as powders from Selleck.cn, were dissolved in dimethyl sulfoxide or water (FLU) to a concentration of 6400ug/ml and stored at – 20°C.

### *In vitro* effect of pyrvinium pamoate alone and combined with azoles or amphotericin B against *Cryptococcus neoformans*

The effects of PP alone, PP–azoles and PP-AmB interactions against *C. neoformans* were evaluated *via* the microdilution chequerboard technique, adapted from broth microdilution method as described in the CLSI M27-A4 (Drogari-Apiranthitou et al., 2012; Clinical and Laboratory Standards Institute, 2017). Inoculum concentrations were adjusted to 1×10^6^ to 5×10^6^ CFU/ml ensuring that the final concentration in the 96-well plates was 0.5×10^3^ to 2.5×10^3^ CFU/ml. The final concentrations of PP ranged from 0.25 to 16 µg/ml in rows, the azoles and the AmB concentrations ranged from to 0.03 to16 μg/ml columns. After 72h of incubation at 35°C, the MIC values (MICs) were determined that PP and azoles were read as the lowest concentration required to support 50% growth inhibition compared with the growth in the control wells, and AmB MICs were determined to be at 100% inhibition. A fractional inhibitory concentration index (FICI) value of ≤0.5 indicates synergy, a FICI value of >4 indicates antagonism, and a FICI value of >0.5 and ≤4 indicates no interaction; the FICI was calculated by the formula: FICI = (Ac/Aa) + (Bc/Ba), where Ac and Bc are the MICs of antifungals in combination, and Aa and Ba are the MICs of antifungals A and B alone, respectively (Odds, 2003). All tests were performed in triplicate.

### *In vivo* efficacy of pyrvinium pamoate alone and in combination with azoles or amphotericin B in *Galleria mellonella*

The *in vivo* antifungal activity of PP alone and in combination with azoles and AmB against *C. neoformans* infections was evaluated by *G. mellonella* survival assay as described previously (Maurer et al., 2015). Twenty larvae with sixth instar for every group (∼300 mg, Sichuan, China) were maintained in the dark at room temperature before experiments. Fungal cultures of G7 were grown on PDA at 37°C for 48 h. Yeast were adjusted to 1 × 10^6^ CFU/ml in sterile saline. For evaluation of the *in vivo* effects of PP alone and combined with azoles or AmB, the following intervention groups were included: PP group, ITR group, VOR group, POS group, FLU group, PP with ITR group, PP with VOR group, PP with POS group, and PP with FLU group, AmB group, PP with AmB group, and three control groups including untreated control group, saline control group and yeast control group. The concentrations of drugs were 200 mg/l. A total of 10 μl yeast suspension or saline were injected into the larvae *via* the last right proleg using a Hamilton syringe (25 gauge, 50 μl).

Larvae were infected with fungal suspension 2 h before introducing therapeutic agents 5 μl (1μg/worm). All groups of larvae were incubated at 37°C in the dark. For survival studies, the death of larvae was monitored by visual inspection of the color (brown-dark/brown) every 24 h for a duration of 6 days. The experiment was repeated triplicate using larvae from different batches.

### Statistical analysis

Data were presented as mean ± SEM. Graph Pad Prism 7 was used for graphs and statistical analyses. The survival curves were analyzed by the Kaplan–Meier method. Differences were considered significant when *P* < 0.05.

## Results

### *In vitro* effect of pyrvinium pamoate alone and combined with azoles or AmB against *Cryptococcus neoformans*

The MICs, MIC_50_s, MIC_90_s, geometric mean (GM) and FICI for PP alone and combinations with azoles or AmB against the *C. neoformans* are presented in **Table 1**. The MICs of PP against all isolates were>16 μg/ml. The MIC ranges of ITR were 0.25-4 μg/ml, of VOR were 0.06-1 μg/ml, of POS were 0.125-1 μg/ml, of FLU were 1-16 μg/ml, and of AmB were 0.25-2 μg/ml against all isolates. When PP was combined with ITR, VOR, POS, FLU or AmB, synergism was observed in 13 (65.0%, FICI 0.188–0.365), 3 (15.0%, FICI 0.245-0.301), 19 (95.0%, FICI 0.188-0.375), 7 (35.0%, FICI 0.188-0.375), and 12(60.0%, FICI 0.281-0.375) isolates of *C. neoformans*, respectively (**Table 1**). The MIC_50_s, MIC_90_s, GM of PP drop from all above 16 μg/ml to 4 μg/ml, 4.4 μg/ml and 3.95 μg/ml when combined with ITR; 2 μg/ml, 4 μg/ml and 2.6 μg/ml with VOR; 4 μg/ml, 5 μg/ml and 3.55 μg/ml with POS; 4 μg/ml, 4.4 μg/ml and 2μg/ml with FLU; 4 μg/ml, 4 μg/ml and 3.65 μg/ml with AmB. There was no antagonism observed in all combinations.

**Table 1:** Susceptibility of azoles alone and in combination with Pyrvinium Pamoate against *Cryptococcus neoformans*.

Strains	MIC (µg/ml)										
	Agent alone						Combination (FICI)^a^				
	PP	ITR	VOR	POS	FLU	AMB	PP/ITR	PP/VOR	PP/POS	PP/FLU	PP/AMB
Z1	>16	0.25	0.06	0.5	4	0.25	4/0.06 (0.365, S)	1/0.06 (1.031, I)	2/0.125 (0.313, S)	4/0.25 (0.188, S)	4/0.06 (0.365, S)
Z2	>16	0.5	0.125	1	8	0.25	8/0.125 (0.5, I)	1/0.125 (1.031, I)	2/0.125 (0.188, S)	4/8 (1.125, I)	4/0.06 (0.365, S)
Z3	>16	0.5	0.125	1	8	0.5	8/0.25 (0.75, I)	2/0.06 (0.543, I)	4/0.25 (0.375, S)	4/8 (1.125, I)	4/0.25 (0.625, I)
G5	>16	1	0.25	0.25	8	1	4/0.125 (0.25, S)	4/0.03 (0.245, S)	4/0.06 (0.365, S)	4/8 (1.125, I)	4/0.25 (0.375, S)
G6	>16	0.5	0.125	0.5	2	0.5	4/0.06 (0.245, S)	2/0.03(0.303, S)	2/0.125 (0.313, S)	8/1 (0.75, I)	4/0.25 (0.625, I)
G7	>16	4	1	1	16	1	4/0.25 (0.188, S)	2/0.25 (0.313, S)	4/0.25 (0.375, S)	4/4 (0.375, S)	4/0.25 (0.375, S)
G8	>16	0.5	0.25	0.5	8	0.5	2/0.125 (0.313, S)	2/0.25 (1.063, I)	2/0.25 (0.563, I)	4/8 (1.125, I)	2/0.125 (0.313, S)
G9	>16	0.5	0.125	0.5	16	0.5	4/0.5 (1.125, I)	4/0.125 (1.125, I)	4/0.125 (0.375, S)	4/8 (0.625, I)	4/0.125 (0.375, S)
G10	>16	1	0.25	0.5	4	2	4/0.125 (0.25, S)	4/0.125 (0.625, I)	4/0.125 (0.375, S)	4/4 (1.125, I)	4/0.5 (0.375, S)
08026	>16	0.25	0.125	0.5	8	0.25	4/0.125 (0.625, I)	2/0.06 (0.543, I)	2/0.125 (0.313, S)	8/8 (1.25, I)	4/0.25 (1.125, I)
05781	>16	0.5	0.125	0.5	4	0.5	4/0.06 (0.245, S)	2/0.06 (0.543, I)	4/0.06 (0.245, S)	4/4 (1.125, I)	4/0.25 (0.625, I)
08061	>16	0.5	0.06	0.25	1	1	4/0.06 (0.245, S)	2/0.03 (0.563, I)	4/0.03 (0.245, S)	4/0.5 (0.625, I)	4/0.5 (0.625, I)
07789	>16	0.25	0.06	0.125	1	0.25	1/0.125 (0.531, I)	4/0.03 (0.625, I)	4/0.03 (0.365, S)	2/0.25 (0.313, S)	4/0.25 (1.125, I)
07406	>16	0.5	0.125	0.5	8	0.5	2/0.25 (0.563, I)	2/0.125 (1.063, I)	4/0.125 (0.375, S)	4/8 (1.125, I)	2/0.125 (0.313, S)
07716	>16	0.25	0.06	0.5	4	0.5	4/0.06 (0.365, S)	2/0.03 (0.563, I)	4/0.06 (0.245, S)	4/4 (1.125, I)	1/0.125 (0.281, S)
07109	>16	0.25	0.125	0.5	4	0.5	4/0.125 (0.625, I)	2/0.06 (0.543, I)	2/0.125 (0.313, S)	4/1 (0.375, S)	4/0.5 (1.125, I)
05338	>16	0.25	0.06	0.5	4	0.25	2/0.06 (0.301, S)	4/0.06 (1.125, I)	2/0.125 (0.313, S)	4/1 (0.375, S)	4/0.125 (0.625, I)
06090	>16	0.25	0.125	0.5	4	0.25	4/0.06 (0.365, S)	4/0.06 (0.605, I)	4/0.125 (0.375, S)	4/1 (0.375, S)	4/0.06 (0.365, S)
05009	>16	0.25	0.06	0.5	8	2	4/0.03 (0.365, S)	4/0.06 (1.125, I)	4/0.125 (0.375, S)	4/1 (0.25, S)	4/0.5 (0.375, S)
07394	>16	0.5	0.06	0.5	8	2	4/0.06 (0.245, S)	2/0.06 (1.063, I)	4/0.06 (0.245, S)	2/4 (0.563, I)	4/0.5 (0.375, S)
MIC_50_s	>16	0.5	0.125	0.5	6	0.5	4/0.125	2/0.06	4/0.125	4/4	4/0.25
MIC_90_s	>16	1	0.25	1	8.8	2	4.4/0.25	4/0.138	5/0.25	4.4/8	4/0.5
GM	>16	0.625	0.165	0.531	6.4	0.725	3.95/0.132	2.6/0.085	3.55/0.121	2/3.95	3.65/0.253

PP, pyrvinium pamoate; FLU, fluconazole; ITR, itraconazole; VOR, voriconazole, POS, posaconazole; AmB, amphotericin; MIC, minimal inhibitory concentration; GM, geometric mean.

a FICI, fractional inhibitory concentration index; S, synergy (FICI of ≤0.5); I, no interaction (indifference; 0.5 < FICI ≤ 4). For FICI calculations, concentrations of 32 μg/ml were used when MICs were >16 μg/ml. (The off-scale MICs had been converted to the next highest 2-fold dilution for calculating FICIs).

### *In vivo* efficacy of pyrvinium pamoate alone and in combination with azoles or AmB against *Cryptococcus neoformans*

**Figure 1:**
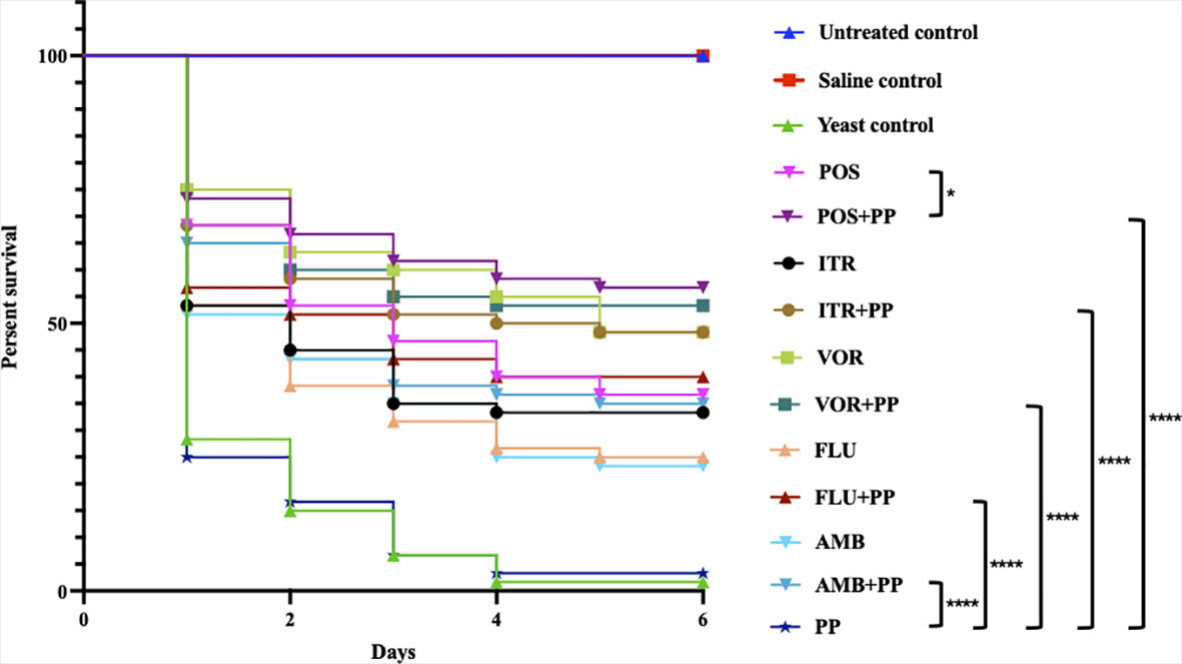
*Galleria mellonella* survival curves following infection with *Cryptococcus neoformans*. Untreated group, uninfected larvae; Saline group, larvae injected with saline; yeast group, larvae infected with *C. neoformans* without any treatment; POS, *C. neoformans*-infected larvae treated with posaconazole (POS) alone; POS+PP, *C. neoformans*-infected larvae treated with POS combined with pyrvinium pamoate (PP); ITR, *C. neoformans*-infected larvae treated with itraconazole alone; ITR+PP, *C. neoformans*-infected larvae treated with ITR combined with PP; VOR, *C. neoformans*-infected larvae treated with voriconazole alone; VOR+PP, *C. neoformans*-infected larvae treated with VOR combined with PP; FLU, *C. neoformans*-infected larvae treated with fluconazole alone; FLU+PP, *C. neoformans*-infected larvae treated with FLU combined with PP; AmB, *C. neoformans*-infected larvae treated with amphotericin B alone; AmB+PP, *C. neoformans*-infected larvae treated with AmB combined with PP; PP, *C. neoformans*-infected larvae treated with PP alone (*p< 0.05. ****p< 0.0001).

The survival rates of larvae treated with PP (3.33%) showed almost no antifungal effective, the survival curve is consistent with the yeast control group (**Figure 1**). The survival rates of larvae for single AmB (23.33%), FLU (25%), ITR (33.33%), POS (36.67%) and VOR (48.33%) group showed less than 50%, but when PP combined with them, they were increased to 35% (PP+AMB), 40% (PP+FLU), 48.33% (PP+ITR), 56.67% (PP+POS), and 53.33% (PP+VOR). Especially, the survival rate for the PP+POS combination was significantly increasing compared to POS treatment alone with statistically differences (*P* = 0. 04). Also, the survival rates of all of the combination groups were significantly increasing compared with PP treatment alone (*P* < 0.000).

## Discussion

Fungal infections are emerging as critically important threats to global health. Since continuously, requirement of prolonged treatment regimens, the limited selection of clinically effective, nontoxic antifungal therapeutic options have plagued scientists for decades. However, recent years, the evolution of drug resistant strains, and emergence of new pathogens presents a new challenge (Bongomin et al., 2017; Perfect, 2017). For *Cryptococcus* infections, these problems are even more acute, especially in patients suffering from HIV/AIDS. With regional differences, treatment of cryptococcosis was limited by the access to first-line antifungal drugs and increasing rates of antifungal resistance, which results the rates of morbidity and mortality were increasing in immunocompromised individuals (Rajasingham et al., 2017). So, exploring new treatment options against *Cryptococcus* infections which were more effective and less toxic side-effect, and even reduce the resistance evolution of strains, will improve morbidity and mortality rates of cryptococcosis.

In the present study, the *in vitro* antifungal tests showed MICs of PP against *C. neoformans* were relatively high, consider with the survival rates of larvae treated with PP *in vivo*, PP alone exerts no inhibition effective against *C. neoformans* unlike the antifungal effective in *C. albicans*, *A. fumigatus* and *E. dermatitidis* (Chen et al., 2015; Gao et al., 2018; Sun et al., 2020a; Sun et al., 2020b). But when PP combined with the commonly used antifungal drugs *in vitro*, synergy effects were detected in all combinations, especially with POS, synergism up to 95.0%, just one isolate showed no interaction. FLU, as one of the mainly widespread drugs against cryptococcosis in many regions, 35.0% isolations show synergy effects. Also, the first line antifungal AmB, often combined with FLU or 5FC in clinical settings, when combined with PP, synergy effects were found in most isolates (60.0%). According to the *in vivo* survival assays, the *in vitro* data were confirmed due to PP combined with azoles and AmB improved larvae survival, especially when PP combined with POS, the larvae survival rate significantly increase (P =0.04).

Recent years, PP has been found to be a potent inhibitor of tumor cells including pancreas, colon, breast, brain, myeloma, and other hematological malignancies. Its inhibition for tumor cells not only selectively that means not affecting the normal/healthy cells of the body, but also with different mechanisms among the tumor types (Esumi et al., 2004; Harada et al., 2012; Momtazi-Borojeni et al., 2018). By inhibiting the Wnt/β-catenin pathway and mitochondrial activity were the most closely watched research hotspot. But, for PP’s fungistatic, the underlying mechanisms remain unclear.

In the present study, PP exhibited no inhibition for *C. neoformans* alone *in vitro* and *in vivo*, but the combinations showed synergy effects, we speculate aneuploidy and its related evolutionary trap (ET) participated in this process. Aneuploidy is a genomic state due to the gain or loss of chromosomes, and the eradication of aneuploids *via* dual-stress application means ET (Torres et al., 2008). Previous studies had demonstrated PP could strongly inhibit the growth of the aneuploidy-based azole resistance *C. albicans* strain i(5L) (Selmecki et al., 2006; Chen et al., 2015), and aneuploidy formation has been found appears to be associated with FLC resistance in *C. neoformans*. Previously studies suggest endoplasmic reticulum (ER) play an important role in the aneuploidy formation under azole stress that ER-associated genes in *C. neoformans* appears amplification under azole stress, although the mechanism of how ER influences aneuploidy formation remains unknown (Kwon-Chung and Chang, 2012). The ER integrity is essential for fungal cells due to ergosterol is produced in the ER and then delivered to plasma membrane, but PP has been proved that can suppress the unfolded protein response (UPR) through glucose starvation to exert anti-tumor activity and play a role in synergistic effective when combined with other drugs (Yu et al., 2008; Ishii et al., 2012; Krishnan and Askew, 2014). In fungal, the UPR is an adaptive signaling pathway, its activation can help eukaryotic cells to adapted ER stress, which protect the fungus from the adverse conditions including antifungal drugs encountered in the host environment and increase the survival of fungal (Sullivan et al., 2006). So, same as tumor cells, PP may disturb the UPR, and then interfere fungi’s ER stress response, combined the stress from antifungals, the pathogenicity and drugs response were weakened or even withdrawn. However, further investigations are needed to elucidate the underlying mechanism.

In summary we show here that PP as an anthelmintic agent, with tolerated and cross the blood–brain barrier, enhance the *in vitro* and *in vivo* activity of POS against *C. neoformans*. This study provides an example of a drug repurposing strategy that PP can be used in the treatment of *C. neoformans* in the future.

## Data availability statement

The original contributions presented in the study are included in the article/supplementary material. Further inquiries can be directed to the corresponding authors.

## Author contributions

YL and JX conceived and designed the study. YL performed all the experiments. YL and SL analyzed the data and wrote the manuscript. MC and HF provided general guidance and revised the manuscript. All authors contributed to the article and approved the submitted version.
